# AI-assisted fetal heart monitoring: a CTG classification model combining attention mechanism and convolutional neural networks

**DOI:** 10.3389/fmed.2026.1804810

**Published:** 2026-04-17

**Authors:** Xinhao Wang, Qingshan You, Tianxin Qiu, Xinghe Zhou

**Affiliations:** Faculty of Science, Civil Aviation Flight University of China, Chengdu, Sichuan, China

**Keywords:** AI-assisted decision-making, computer vision, deep learning, fetal health, fetal heart monitoring

## Abstract

**Objective:**

To develop a deep-learning-based computer vision approach for fetal heart rate (FHR) monitoring that can efficiently detect fetal hypoxia without relying on complex feature extraction methods.

**Methods:**

A hybrid attention mechanism was proposed for direct processing of fetal monitoring images (cardiotocography, CTG), eliminating the need for manual feature extraction. The method leverages deep learning to classify fetal health states based on real-time CTG images.

**Results:**

Experiments on a real-world clinical dataset demonstrated that the proposed method achieved a classification accuracy of 97.94%, indicating its high efficiency in detecting fetal hypoxia.

**Conclusion:**

The proposed hybrid attention-based deep learning approach provides reliable support for the early detection of fetal hypoxia, overcoming the limitations of traditional machine learning methods that rely on complex feature extraction.

## 1 Introduction

The World Health Organization (WHO) and the United Nations Children’s Fund (UNICEF), based on the 2018 global health monitoring data, jointly pointed out that the global maternal and infant health sector still faces severe and urgent challenges that need to be addressed. Data shows that each year, approximately 5.3 million children under the age of five die from health problems such as pneumonia, diarrhea, and complications from premature birth, which could be avoided through basic medical intervention. Among them, the death rate of newborns (within 28 days after birth) accounts for nearly 50%, which fully highlights the critical gap in health monitoring and care services during the neonatal period - children at this stage have not fully developed their physiological functions and have extremely low tolerance to risks such as hypoxia and infection. Without timely intervention, they are prone to develop into fatal health crises. From a regional distribution perspective, the differences in maternal and infant health among regions with different economic development levels are extremely significant, with the situation in sub-Saharan Africa being the most prominent. The maternal mortality rate in this region is nearly 50 times higher than that in high-income economies, and the mortality rate of newborns within 28 days after birth is nearly 10 times that of high-income regions. In terms of long-term survival of children, in 2018, one out of every 13 children in sub-Saharan Africa did not survive to the age of five, a rate 15 times higher than that in regions with abundant medical resources such as Europe. The uneven distribution of health resources, insufficient coverage of primary medical institutions, and shortage of professional medical staff are the common factors that prevent mothers and infants in this region from obtaining timely prenatal check-ups, intrapartum care, and postpartum monitoring services, which are the core reasons for the persistently high mortality rate (1).

The Electronic Fetal Monitoring (EFM) has been used to detect fetal heartbeat and uterine contraction patterns (2). It is a non-invasive device for assessing the health of the fetus in the uterus. It is easy to operate, inexpensive, and capable of long-term continuous monitoring, so it is widely used in obstetrics (3, 4). Electronic fetal heart monitoring equipment can provide a variety of fetal-related data, including fetal information, uterine information, and fetal movement. Among them, fetal heart rate (FHR) is one of the important indicators for assessing fetal health. By recording the relationship between FHR, fetal movement, and uterine contraction (UC) (5, 6), the status of the fetus in the uterus can be reflected. Doctors and nurses evaluate the fetal condition by observing the FHR and UC curves. However, due to the complexity and variability of the FHR and UC curves, medical staff need to have professional knowledge and rich clinical experience, and the analysis and judgment process is easily affected by subjective factors (7), which may lead to misjudgment. With the development of machine learning algorithms and the accumulation of a large amount of electronic fetal heart monitoring data, it has become more effective to use machine learning algorithms to study the health of the fetus in the uterus (8–10).

Machine learning algorithms usually extract relevant features from FHR signals and classify them (11). This method is relatively complex and requires a longer execution process. However, with the rapid development of deep learning and its successful application in various fields, deep learning methods can more effectively help doctors determine possible health problems of the fetus, such as fetal distress, fetal anemia, fetal infection, congenital heart disease and fetal growth restriction (12). At present, domestic and foreign scholars have studied the intelligent algorithm of FHR curves, mainly based on the extracted feature information for classification research. Georgieva et al. (13) first used a simple feedforward artificial neural network to extract 6 FHR features and combined them with 6 related clinical parameters for classification research. Spilka et al. (14) used random forest and latent class analysis to study and developed a hybrid model to classify fetal heart rate. Cömert et al. (15) used short-time Fourier transform and gray-level co-occurrence matrix to convert images into 8-bit grayscale images, and used related features to identify FHR signals. Liu et al. (16) used CNN-BiLSTM hybrid neural network and enhanced the features of discrete wavelet transform (DWT) to classify FHR signals. The aforementioned fetal heart classification algorithms mainly rely on extracting features from time series signals and then applying machine learning related algorithms for classification research. This study is completely based on image processing methods. First, we extract the fetal heart rate (FHR) curve and uterine contraction (UC) curve in the fetal heart monitoring image and convert them into three-channel color images, marking the FHR curve and UC curve with different colors.

The above-mentioned research has the following shortcomings:

(1) Some studies rely on manual extraction of clinical information on fetal heart rate, lacking intelligence and practicality. Such studies require professionals to manually screen and extract clinical features related to fetal health (such as the amplitude of fetal heart rate variability, the interval between contractions, etc.) from a large amount of EFM data, and then input the features into the model for analysis.
(2) Most studies discard the uterine contraction rate (UC) data and only use fetal heart rate (FHR) as the sole basis for analysis, resulting in an incomplete assessment dimension. The health status of the fetus in utero is the result of the interaction between fetal heart rate and uterine contractions. Uterine contractions, as a direct reflection of changes in the intrauterine environment, their intensity, frequency, and duration all have significant impacts on fetal blood oxygen supply, thereby causing corresponding fluctuations in fetal heart rate. Some existing studies deliberately ignore the uterine contraction rate data to simplify the difficulty of model construction and only use the fetal heart rate as a single indicator for health assessment. This approach severs the intrinsic connection between the two, and fails to fully capture the core logic of the dynamic changes of the fetus in utero.
(3) The accuracy of the analysis models and conclusions proposed by some researchers is relatively low, and cannot meet the requirements of clinical diagnosis and treatment.

To address the existing problems in related research, such as insufficient intelligence, incomplete assessment dimensions, and low accuracy, and in light of clinical needs and technological trends, this paper proposes the following three targeted improvement measures. An earlier version of this article was posted as a preprint prior to peer review (DOI: 10.21203/rs.3.rs-5090931/v1) (17):

(1) De-grid processing of fetal heart rate graphs to simultaneously extract fetal heart rate and uterine contraction data.
(2) Distinguishing and presenting data in a graphical form using color-based differentiation to enhance data readability.
(3) Utilizing computer vision-related methods to directly conduct intelligent analysis on the visualized images.

### 1.1 Our contributions

*Direct processing of raw CTG images*: Unlike many previous studies that rely on manually extracted features or use only fetal heart rate (FHR) signals, our method directly processes raw CTG images, preserving more original visual and structural information.

*Joint utilization of FHR and uterine contraction (UC) signals*: The proposed framework incorporates both FHR and UC information, which allows the model to learn more comprehensive fetal-state representations.

*Hybrid attention-enhanced lightweight architecture*: We introduced a hybrid attention (HA) mechanism into ResNet-18 and demonstrated through ablation experiments that selective insertion of HA in deep layers provides the best performance, improving feature representation while avoiding excessive attention redundancy.

*Systematic ablation study*: We did not simply report the performance of a single model, but also evaluated multiple HA insertion strategies to identify the most effective configuration.

## 2 Materials and methods

### 2.1 CTG interpretation standard

The baseline refers to the average fetal heart rate that fluctuates within 5 beats/min over a 10-min period, excluding acceleration, deceleration, and significant variability. The normal range for the FHR baseline is 110–160 beats/min (18). To be considered a baseline, the graph must last for more than 2 min within any 10-min period, though it can be discontinuous. If the baseline is unclear during the observation phase, it can be determined by referencing the graph from the previous 10 min. Specific conditions include: (1) Fetal Tachycardia: When the fetal heart baseline exceeds 160 beats/min and lasts for at least 10 min. If the baseline is unclear, the previous 10-min graph can be used to establish the baseline; (2) Fetal Bradycardia: When the fetal heart baseline drops below 110 beats/min for a duration of 10 min or more.

**Figure 1:**
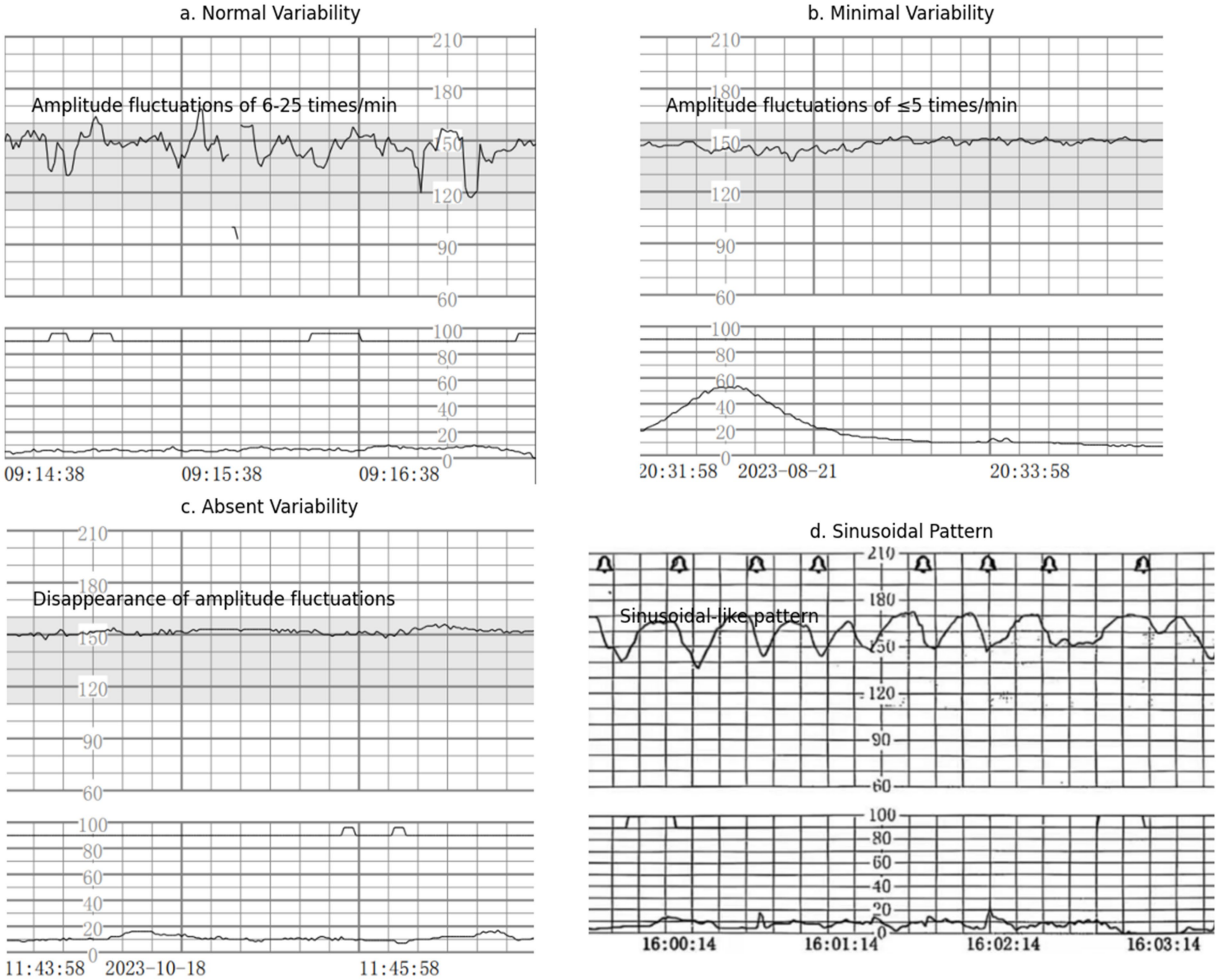
Baseline variation and sinusoidal pattern. **(a)** Normal variability, **(b)** Minimal variability, **(c)** Absent variability, **(d)** Sinusoidal pattern.

**Figure 2:**
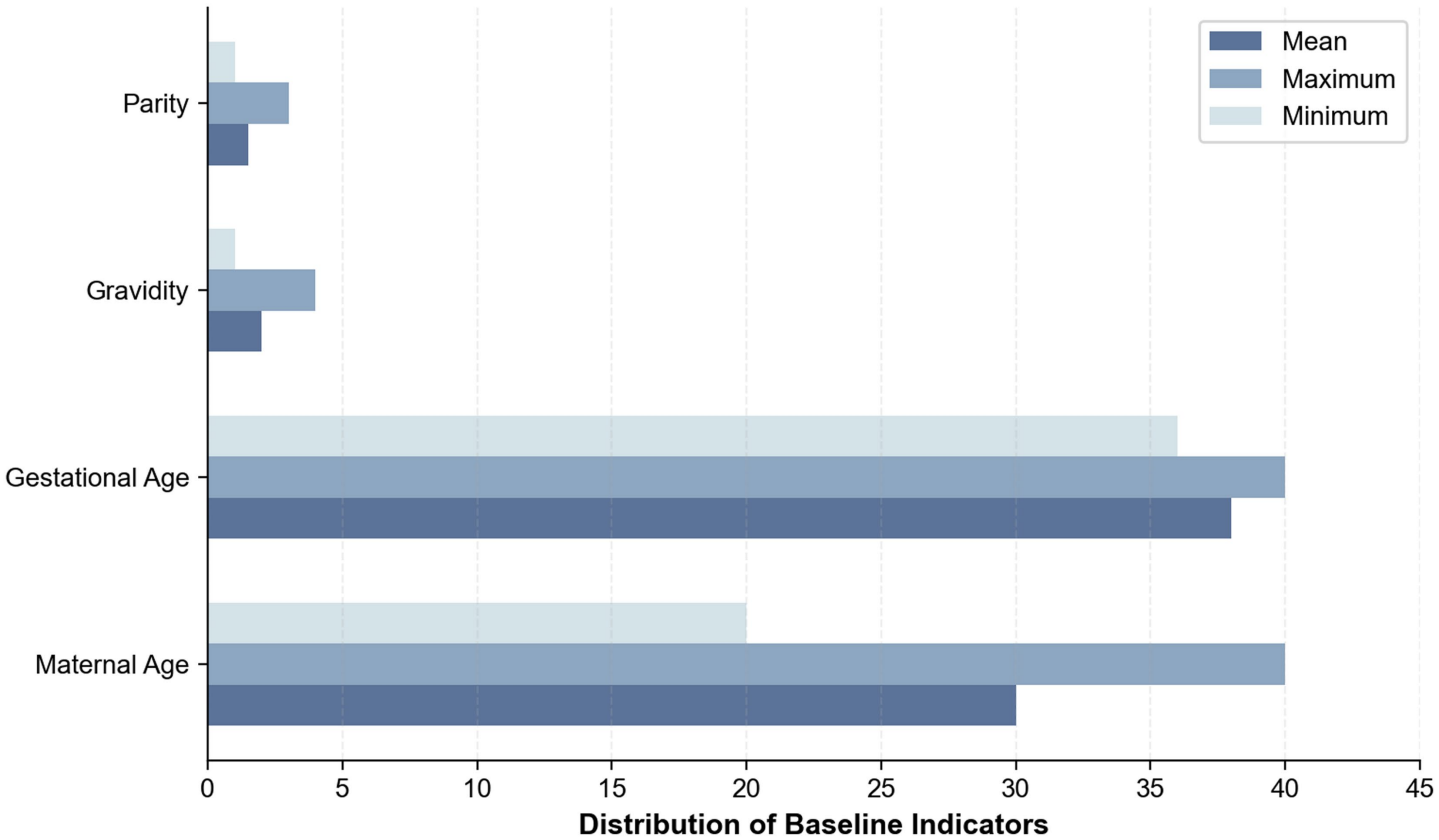
Pregnant woman infographic.

**Figure 3:**
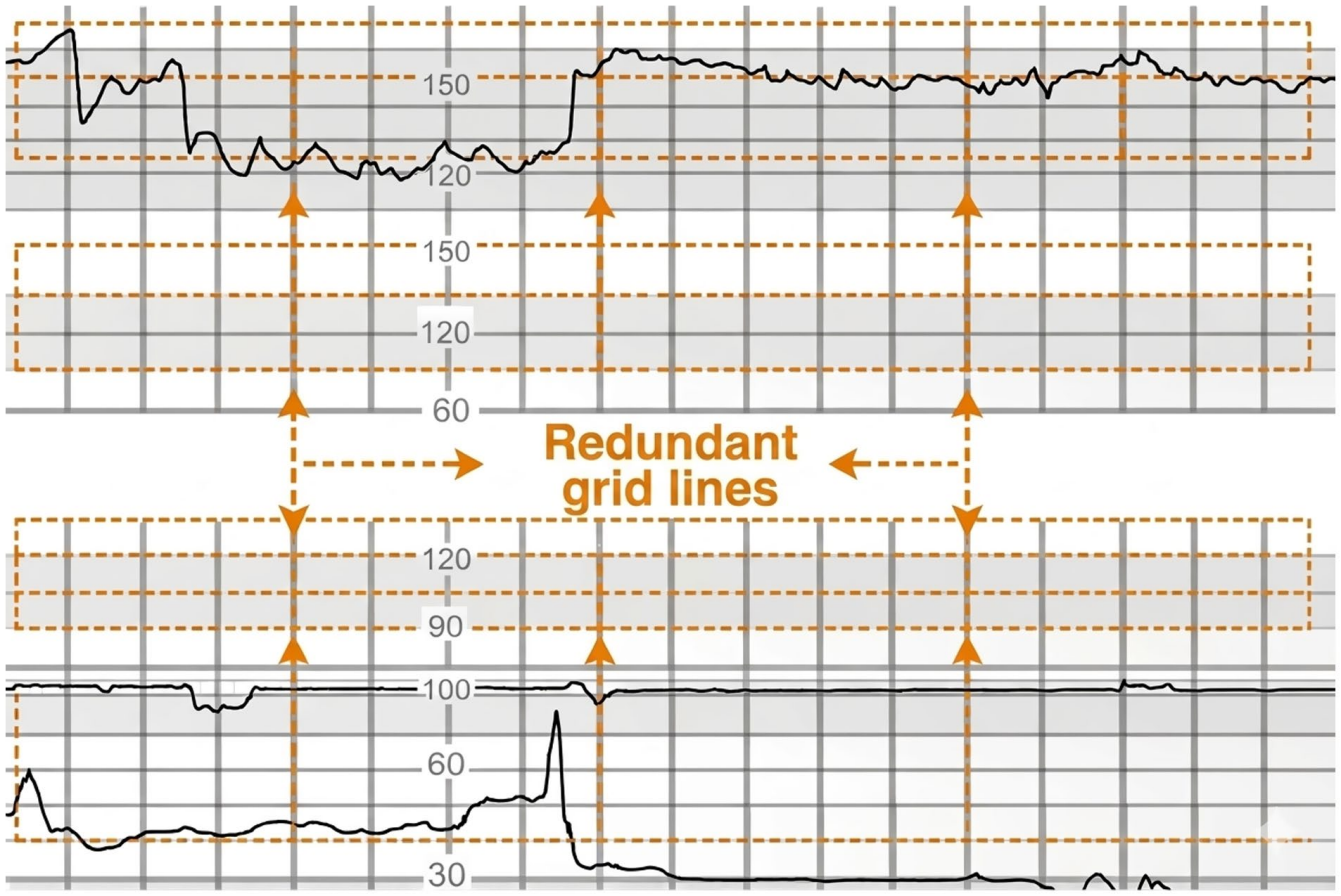
Extraneous grid lines.

Baseline variability refers to the changes in amplitude of the fetal heart rate per minute from the peak to the trough, which can be visualized and quantified (19). Types of variability include: (1) Absent Variability: The complete disappearance of amplitude fluctuations, as shown in Figure 1c; (2) Minimal Variability: Amplitude fluctuations of ≤5 times/min, as shown in Figure 1b; (3) Normal Moderate Variability: Amplitude fluctuations of 6–25 times/min, as shown in Figure 1a.

Acceleration refers to a sudden and significant increase in the baseline fetal heart rate, with a start-to-peak time of less than 30 s. The duration from the start of the acceleration to the return to baseline is known as the acceleration time. Acceleration characteristics are: (1) Before 32 weeks of gestation, acceleration is defined as an increase of ≥10 beats/min above the baseline, lasting for ≥10 s but <2 min. (2) After 32 weeks of gestation, acceleration is defined as an increase of ≥15 beats/min above the baseline, lasting for ≥15 s but <2 min; (3) Prolonged acceleration: When the increase in fetal heart rate lasts for ≥2 min but <10 min.; (4) If acceleration lasts for ≥10 min, the change in baseline fetal heart rate is considered.

The sinusoidal pattern is characterized by a distinct, smooth, sinusoidal-like shape with a long cycle variant of 3–5 cycles per minute (20), lasting for 20 min or more, without any acceleration. This pattern is clearly visible, as shown in Figure 1d.

Deceleration refers to a decrease in fetal heart rate, and can be categorized into three types: (1) Early Deceleration (ED) occurs in conjunction with contractions, usually symmetrical, with a slow decline to the nadir followed by a return to baseline. The time from the onset to the nadir is ≥30 s, and the nadir often coincides with the peak of the contraction. Generally, the onset, nadir, and recovery of the deceleration are synchronized with the onset, peak, and end of the contractions; (2) Late Deceleration (LD) also occurs with contractions, typically symmetrical, with a gradual decrease to the nadir and a return to baseline. The time from the onset to the nadir is ≥30 s, but the nadir is usually delayed from the peak of the contraction. The onset, nadir, and recovery of the deceleration generally lag behind the onset, peak, and end of the contraction, respectively; (3) Variable Deceleration (VD) refers to a sudden, significant, and rapid decline in fetal heart rate, with an onset-to-nadir time of <30 s, a decline of ≥15 beats/min, and a duration of ≥15 s but <2 min. When variable deceleration occurs with contractions, the onset is usually delayed from the peak of the contraction, and there is no fixed pattern between the onset, depth, and duration of deceleration and the contractions.

### 2.2 Datasets

#### 2.2.1 CTU-UHB

The CTU-UHB dataset (jointly released by the Czech Technical University and the University Hospital Brno) (21) is widely used as a benchmark in the field of fetal heart rate monitoring research. This dataset is based on 9,164 original cardiotocography (CTG) records collected between 2010 and 2012, with 552 samples retained after screening, each annotated with complete clinical information. Although the dataset’s scale and openness provide convenience for algorithm development, several key limitations still exist, which restrict its practical value in clinical applications. This dataset has several drawbacks:

(1) The CTU-UHB dataset has a significant racial homogeneity, covering only European Caucasian pregnant women, resulting in highly homogeneous fetal heart rate (FHR) and uterine contraction (UC) signal characteristics. However, the target application scenario involves Chinese pregnant women whose physiological dynamics may be influenced by factors such as geographical environment and racial genetic background, exhibiting group-specific patterns, such as systematic shifts in baseline heart rate and differences in physiological responses to uterine contraction stress. This inherent bias in data distribution poses a significant challenge to the transfer application of models trained on single-race samples to heterogeneous populations, thereby inducing cross-population clinical decision bias and reducing the model’s adaptability and reliability in the target scenario.
(2) The dataset suffers from severe missing or incomplete uterine contraction signals. This data limitation forces related studies to be limited to the analysis of a single fetal heart rate channel, fundamentally deviating from the core guidelines of “dual-parameter collaborative assessment” in clinical practice. In clinical diagnosis and treatment, accurate assessment of fetal physiological status relies on the time-frequency domain coupling feature analysis of fetal heart rate and uterine contraction signals. For example, early warning signals of fetal hypoxia often manifest as the synergistic appearance of increased uterine contraction pressure and the absence of fetal heart rate acceleration response. However, a single fetal heart rate modality cannot capture such multi-dimensional dynamic correlation information, thus limiting the model’s ability to identify key clinical pathological states.

#### 2.2.2 Datasets used

To overcome the adaptability limitations of publicly available datasets in regional clinical applications, this study, in collaboration with the obstetrics center of a top-tier hospital in Chengdu, constructed a dedicated dataset for Chinese pregnant women. This dataset consecutively included 489 singleton pregnancies who underwent routine fetal heart rate monitoring at the obstetrics outpatient clinic of the First People’s Hospital of Longquanyi District, Chengdu, Sichuan Province, China, from March 2023 to March 2025. Pregnancy information is shown in Figure 2. Cases with severe fetal malformations, maternal arrhythmias, or signal loss exceeding 5 min were excluded. The data was independently annotated by two obstetricians with ≥5 years of experience according to the FIGO 2015 guidelines; disagreements were arbitrated by a third party. The data distribution was as follows: 224 normal cases and 265 pathological cases. The pathological cases in this study were not defined by a single disease entity, but by clinically abnormal CTG patterns according to the FIGO 2015 interpretation criteria. Specifically, these recordings mainly included one or more non-reassuring or pathological features, such as persistent fetal tachycardia or bradycardia, absent or minimal baseline variability, recurrent late decelerations, recurrent variable decelerations, prolonged decelerations, and sinusoidal patterns. Therefore, the pathological group represented a clinically heterogeneous set of abnormal fetal monitoring recordings rather than a single specific obstetric complication.

The CTU-UHB dataset suffers from a significant data distribution imbalance. Its criterion for classifying fetal status as normal or abnormal relies primarily on the pH value of fetal umbilical cord blood. Regardless of the pH threshold used, the resulting dataset consistently exhibits a significantly larger number of normal samples than abnormal samples. In contrast, the dataset used in this study demonstrates a more balanced distribution of normal and abnormal samples. This feature effectively avoids the modeling drawbacks caused by data imbalance and better highlights the superiority and suitability of the dataset chosen for this study in terms of sample distribution.

#### 2.2.3 Datasets preprocessing

In fetal heart rate monitoring image analysis, grid lines often interfere with the extraction of fetal heart rate (FHR) signals, as shown in Figure 3. To improve the accuracy and usability of the signal, this study proposes a template matching-based fetal heart rate extraction algorithm to remove grid line background interference from fetal heart rate images. This algorithm utilizes template matching technology to identify and remove periodic grid lines in the image, thereby achieving accurate extraction of the fetal heart rate signal.

Each image has a size of M×W , where M is the image height, and W is the image width. The pixel intensity values of the image are defined as $I\left(\mathrm{x,y}\right)\in \left[0,255\right].$ By setting the threshold value T , the grayscale image is converted into a binary image B(x,y) as shown in Equation (1):


$$B\left(x,y\right)=1-\{\begin{array}{ll} 1, & I\left(x,y\right)>T\\ 0, & I\left(x,y\right)\leq T \end{array}$$ (1)

Where T=50 , The threshold T = 50 was determined through preliminary experiments define the FHR template $T_{f}\left(i\right)$ and the shrinkage template $T_{u}\left(i\right)$ , which are generated based on line scan radiation as shown in Equation (2):


$$\begin{array}{l} T_{f}\left(i\right)=270-\mathrm{linspace}\left(60,210,N_{f}\right),\;\\ i=1,2,\ldots ,N_{f} \end{array}$$ (2)

Where $N_{f}$ and $N_{u}$ represent the vertical pixel intensity values of the FHR and UC, respectively. $T_{f}$ and $T_{u}$ are reference templates used for the subsequent region matching. Then, the corresponding FHR and UC in the image are segmented, and four specific regions are defined. For any given Y , the region pixel values are extracted as shown in Equation (3):


$$A_{k}\left(x,y\right)=\left\{\begin{array}{ll} B\left(x,y\right), & \mathrm{if}\;x\in \left[h_{2k-1},h_{2k}\right]\\ 0, & \mathrm{otherwise} \end{array}\right.$$ (3)

Where k=1,2,3,4 represents different specific regions. Then, $A_{k}\left(\mathrm{x,y}\right)$ is extracted and the template $T_{k}\left(x\right)$ is correlated to calculate the feature values. For each row y , the calculation formula for the feature value $F_{k}\left(y\right)$ is shown in Equation (4):


$$F_{k}\left(y\right)=\{\begin{array}{ll} \frac{\underset{x\in \left[h_{2k-1},h_{2k}\right]}{\sum }A_{k}\left(x,y\right)\cdot T_{k}\left(x\right)}{\underset{x\in \left[h_{2k-1},h_{2k}\right]}{\sum }A_{k}\left(x,y\right)}, & \mathrm{if}\;\underset{x\in \left[h_{2k-1},h_{2k}\right]}{\sum }A_{k}\left(x,y\right)>0\\ 0, & \mathrm{otherwise} \end{array}$$ (4)

Where $T_{k}\left(x\right)\;$ represents FHR or UC, $k\in \left\{1,2,3,4\right\}$ . For each image, the feature values $F_{1}\left(y\right),F_{2}\left(y\right),F_{3}\left(y\right),F_{4}\left(y\right)$ are listed. Based on the storage, construct the feature vector X as shown in Equation (5):


$$X=\left[\begin{array}{cccc} F_{1}^{\left(1\right)}\left(1\right) & F_{1}^{\left(1\right)}\left(2\right) & \cdots & F_{1}^{\left(1\right)}\left(W\right)\\ F_{2}^{\left(1\right)}\left(1\right) & F_{2}^{\left(1\right)}\left(2\right) & \cdots & F_{2}^{\left(1\right)}\left(W\right)\\ \vdots & \vdots & \ddots & \vdots \\ F_{4}^{\left(N\right)}\left(1\right) & F_{4}^{\left(N\right)}\left(2\right) & \cdots & F_{4}^{\left(N\right)}\left(W\right) \end{array}\right]$$ (5)

**Figure 4:**
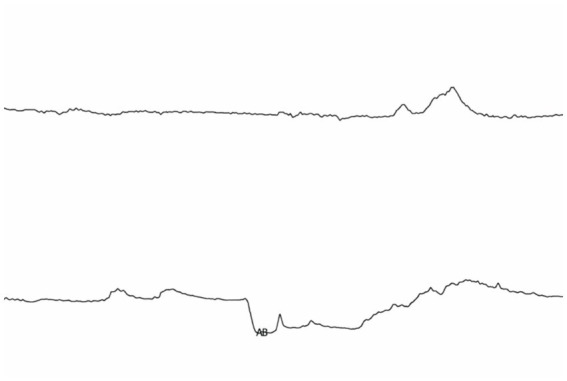
The fetal heart rate graph without grid lines.

Remove grid lines as shown in Figure 4.

The pseudo code is shown in Table 1.

**Table 1:** Preprocessing pseudo code.

Algorithm 1: Template-guided signal extraction from CTG images
Input: CTG image set I, binary threshold T Output: Sequence matrix X
1: Read all CTG images and convert them to grayscale 2: Construct fetal heart rate template FHR and uterine contraction template UC 3: Initialize sequence matrix X 4: for each image in I do 5: Binarize the image using threshold T and invert the binary result 6: for each image column do 7: Extract four predefined signal regions 8: Match fetal heart rate regions with taixin and contraction regions with gongsuo 9: Compute the mean of nonzero matched values for each region 10: Store the four extracted values into X 11: end for 12: end for 13: Export X to Excel

**Figure 5:**
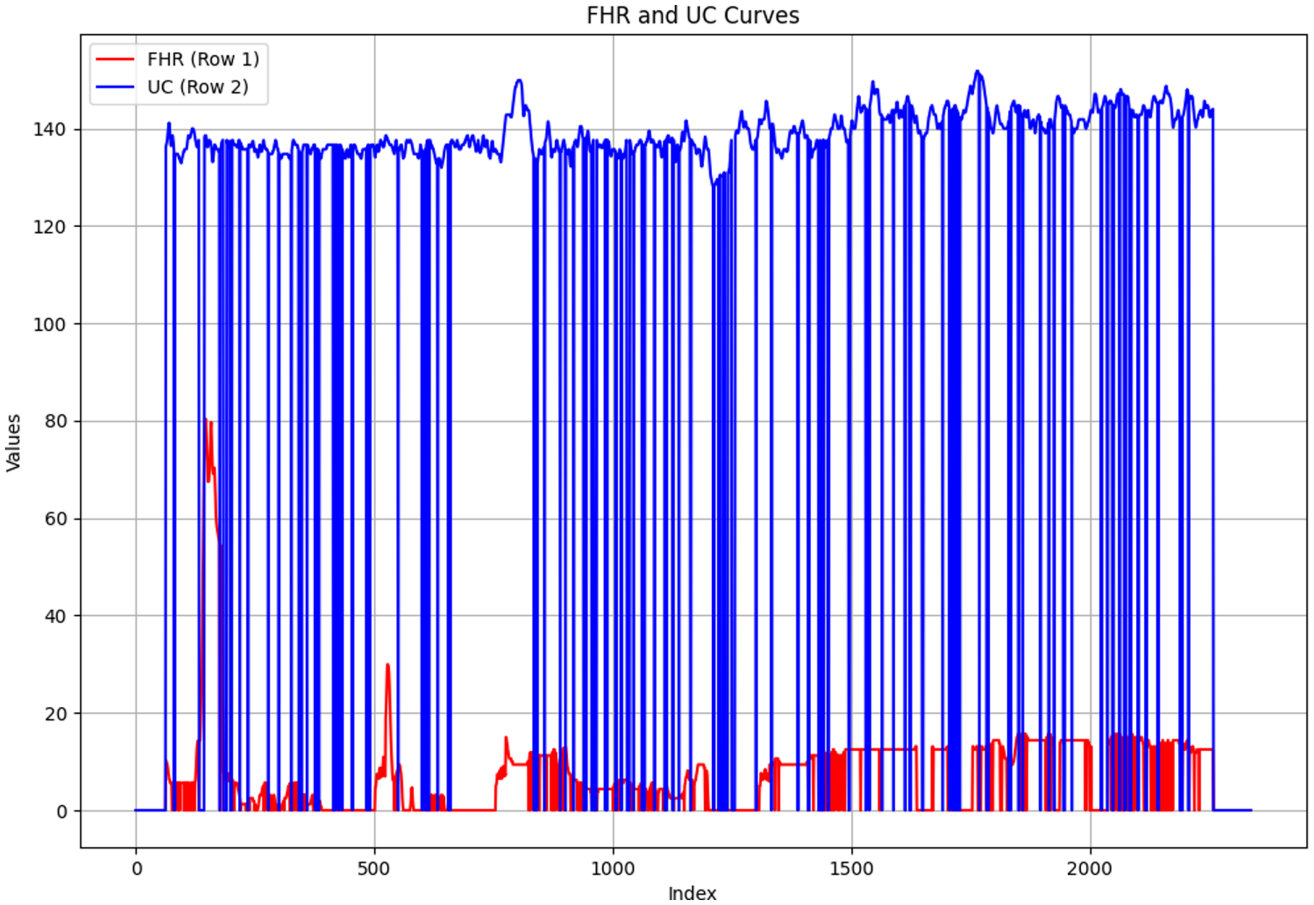
Unprocessed fetal heart signals and uterine contraction signals.

In fetal heart rate monitoring images, after removing grid lines, the extracted fetal heart rate (FHR) and uterine contraction rate (UC) signals often exhibit obvious zeros, discontinuities, and abnormal peak values. These discontinuities and extreme values are usually caused by intermittent power outages, manual pauses, and other objective factors of the fetal heart rate monitor. Due to these factors, the signal exhibits irregular interruptions or abrupt changes in the time series, resulting in severe noise contamination of the original signal. This noise not only reduces the reliability of the signal but also makes subsequent algorithm modeling more complex and difficult. Therefore, before further data analysis and feature extraction, the signal must be processed using appropriate interpolation and denoising methods to restore its smoothness and continuity, providing accurate and reliable input for deep learning models. The original fetal heart rate signal is shown in Figure 5.

While Lagrange interpolation can meet the basic requirement of continuity in fetal heart rate and uterine contraction data interpolation to a certain extent, it has two core problems for processing fetal heart rate data:

(1) Fetal heart rate monitoring data typically has a massive number of discrete sampling points. Directly applying Lagrange interpolation requires constructing a high-order interpolation polynomial, whose time complexity increases exponentially with the number of data points, leading to a significant decrease in data processing efficiency and making it difficult to meet the timeliness requirements of real-time clinical analysis or batch data processing.
(2) Lagrange interpolation is essentially a global polynomial interpolation method. Its fitting process is guided by the overall data distribution, and it lacks the ability to characterize local data features. The core of clinical analysis of fetal heart rate maps lies in capturing the local dynamic trends of fetal heart rate signals (such as instantaneous heart rate fluctuations, heart rate changes associated with uterine contractions, etc.). Global interpolation can easily mask key local features, and even cause distortion of local trends due to Runge’s phenomenon in high-order polynomials.

**Figure 6:**
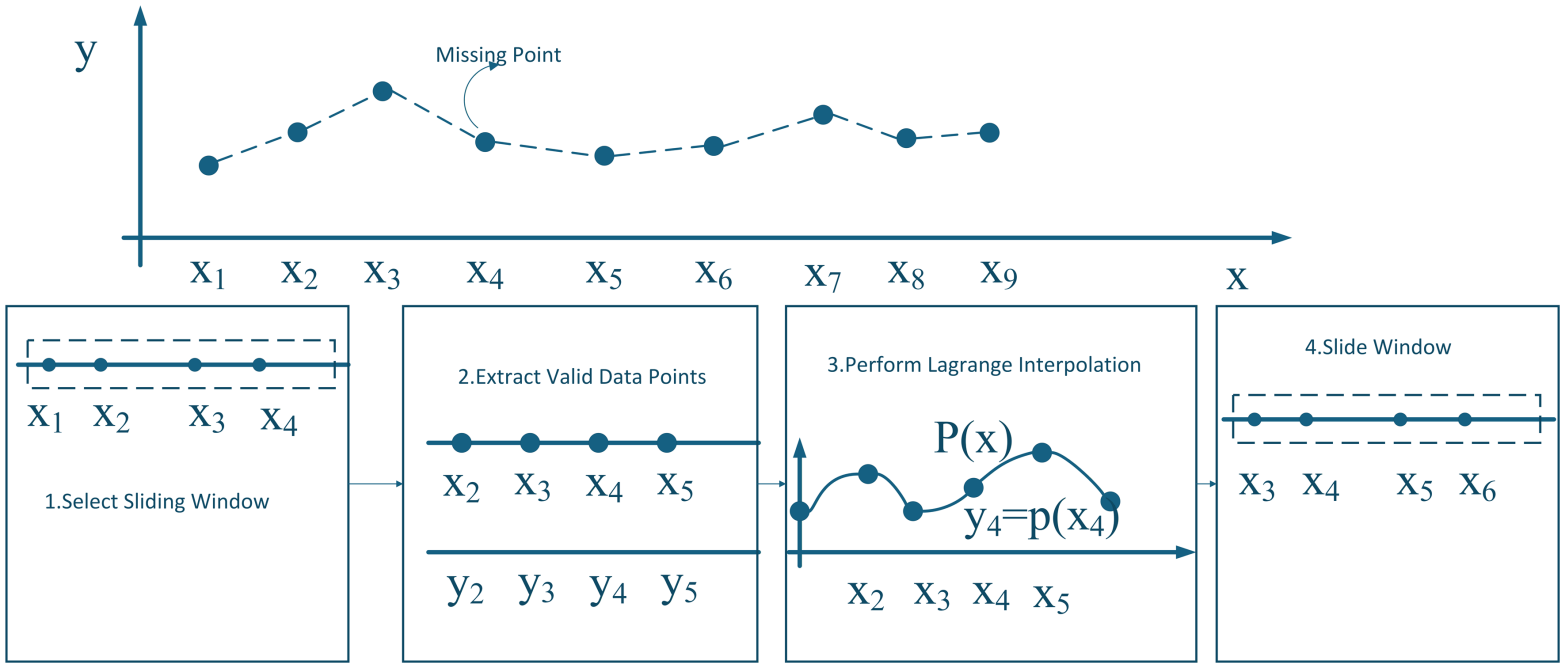
Sliding Window Lagrange interpolation method.

To address the core issues of high time complexity and insufficient local feature characterization in Lagrange interpolation for fetal heart rate and uterine contraction data processing, this paper proposes an improved Lagrange interpolation method based on a sliding window mechanism. This method achieves efficient and accurate local interpolation processing of fetal heart rate and uterine contraction data. The core innovation of this method lies in breaking away from the global fitting mode of traditional Lagrange interpolation. By introducing a dynamic sliding window mechanism, the massive discrete data of fetal heart rate and uterine contractions is decomposed into several overlapping local data subsets. A schematic diagram of the sliding window Lagrange interpolation method is shown in Figure 6.

**Figure 7:**
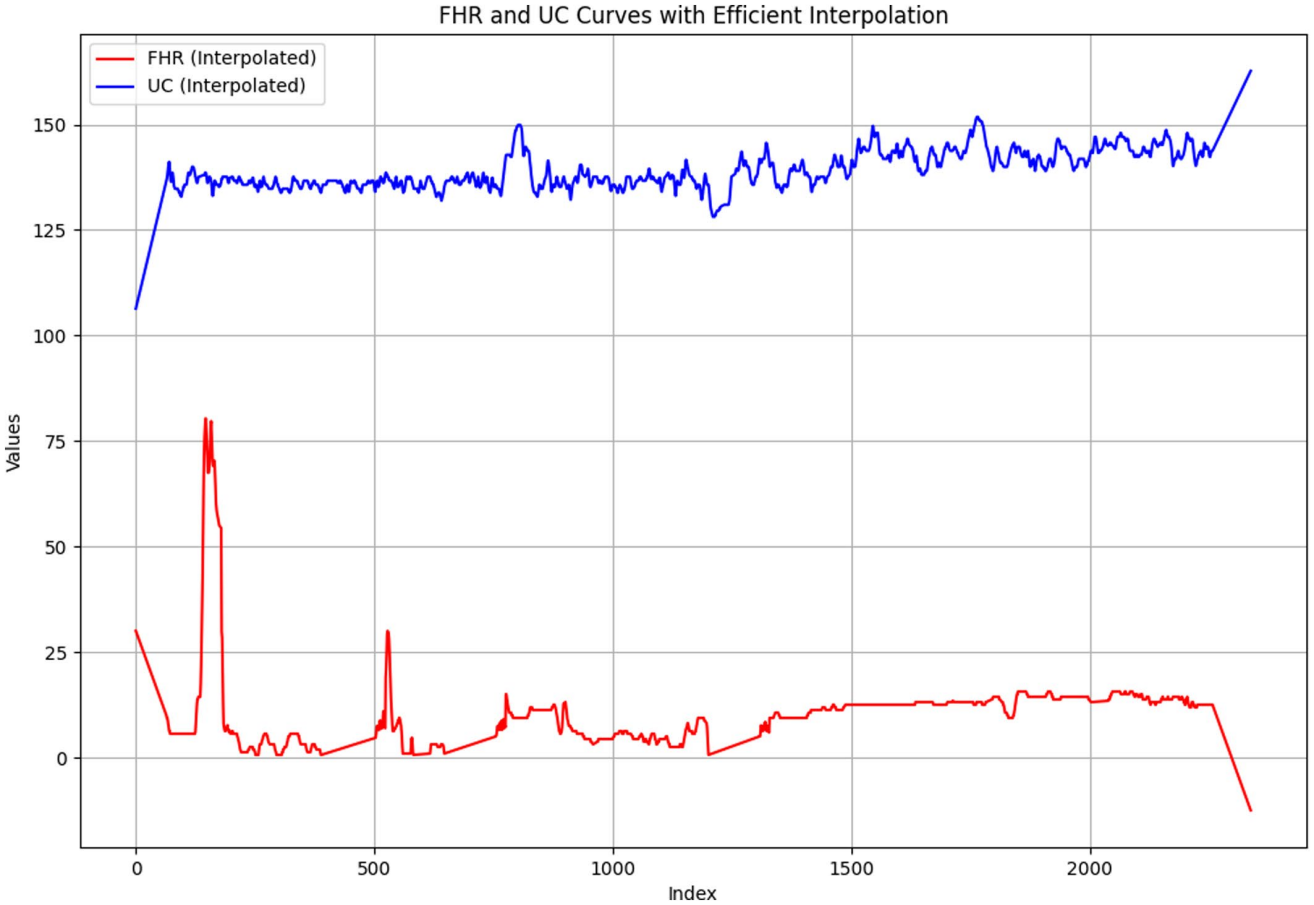
The signal graph after interpolation.

The Sliding Window Lagrange Interpolation algorithm consists of a series of methodical steps aimed at estimating missing data points in a given dataset. Initially, a sliding window is defined to include only valid data points, ensuring that no missing values are present within the selected window. For instance, a set of consecutive data points, such as $x_{1},x_{2},x_{3},x_{4}$ , is chosen, all of which are guaranteed to be valid. Subsequently, valid data points are extracted from the defined window, such as $x_{2},x_{3},x_{5}$ , and the Lagrange interpolation method is employed to estimate the missing value at the target point (e.g., $x_{4}$ ) using these valid points. Following the interpolation process, the window is shifted one step forward, incorporating the next set of data points, for example, $x_{3},x_{4},x_{5},x_{6}$ . This iterative procedure continues until all missing values in the dataset are effectively interpolated. The signal graph after interpolation is shown in Figure 7.

To quantitatively evaluate the effectiveness of the proposed preprocessing pipeline, we performed an additional assessment on representative CTG recordings before and after preprocessing. Because artifact-free pixel-level reference curves were unavailable for the original clinical CTG charts, strict full-reference restoration metrics such as PSNR and MSE could not be directly computed. Therefore, we adopted four practical surrogate indicators that were closely aligned with the objectives of preprocessing.

First, the zero-value rate was calculated as the proportion of zero-valued points in the extracted signal sequence, reflecting the extent of signal interruption or invalid extraction. Second, the interruption segment count was defined as the total number of consecutive zero-value or missing-value segments in a sequence. Third, the mean interruption length was calculated as the average length of all interruption segments, which was used to characterize the severity of discontinuity. Fourth, the expert readability score was independently assigned by two obstetricians using a 5-point Likert scale, where 1 indicated unreadable and 5 indicated excellent readability.

For a signal sequence $x=\left\{x_{1},x_{2},\ldots ,x_{N}\right\}$ , the zero-value rate was calculated as shown in Equation (6):


$$\mathrm{Zero}‐\mathrm{value rate}=\frac{1}{N}\overset{N}{\underset{i=1}{\sum }}1\left(x_{i}=0\right)\times 100\%$$ (6)

Although the template-based de-gridding and sliding-window interpolation were effective for the current dataset, more complex real-world clinical artifacts, such as uneven illumination, handwritten annotations, and severe image distortions, may still affect preprocessing robustness.

An interruption segment was defined as a consecutive sequence of zero-valued or missing points with length ≥1. The interruption segment count was the total number of such segments in each recording, and the mean interruption length was calculated as the average number of points across all interruption segments.

Statistical comparisons between signals before and after preprocessing were performed using the Wilcoxon signed-rank test. A two-sided *p* < 0.05 was considered statistically significant.

We randomly selected fifty CTG recordings for evaluation and analysis, and the results are shown in Table 2.

**Table 2:** Quantitative evaluation of preprocessing effectiveness.

Metric	Before preprocessing	After preprocessing	*p* value
Zero-value rate (%)	12.84 ± 3.17	3.26 ± 1.14	<0.001
Interruption segment count	18.42 ± 4.63	6.18 ± 2.07	<0.001
Mean interruption length	7.95 ± 1.88	2.71 ± 0.93	<0.001
Expert readability score	2.36 ± 0.58	4.21 ± 0.49	<0.001

### 2.3 Attention mechanism

#### 2.3.1 Channel attention mechanism

**Figure 8:**
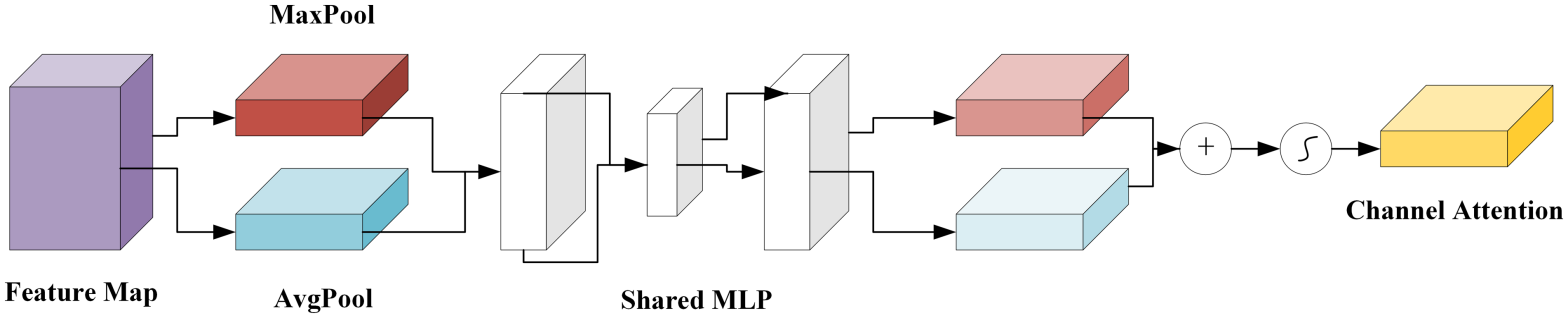
Channel attention mechanism.

Channel Attention Mechanism (CAM) is an effective feature selection method in deep learning. It adaptively adjusts the weights of different channels to enhance key features and suppress redundant information, thereby improving the performance of neural networks in complex tasks. In Convolutional Neural Networks (CNNs), the input feature map is processed through multiple convolutional layers to generate feature maps with multiple channels, each carrying different types of features (e.g., edges, textures). However, not all channels contribute equally to the target task. Therefore, the Channel Attention Mechanism optimizes the network’s focus on key features by assigning different weights to each channel. The process of Channel Attention is shown in Figure 8.

#### 2.3.2 Spatial attention mechanism

**Figure 9:**
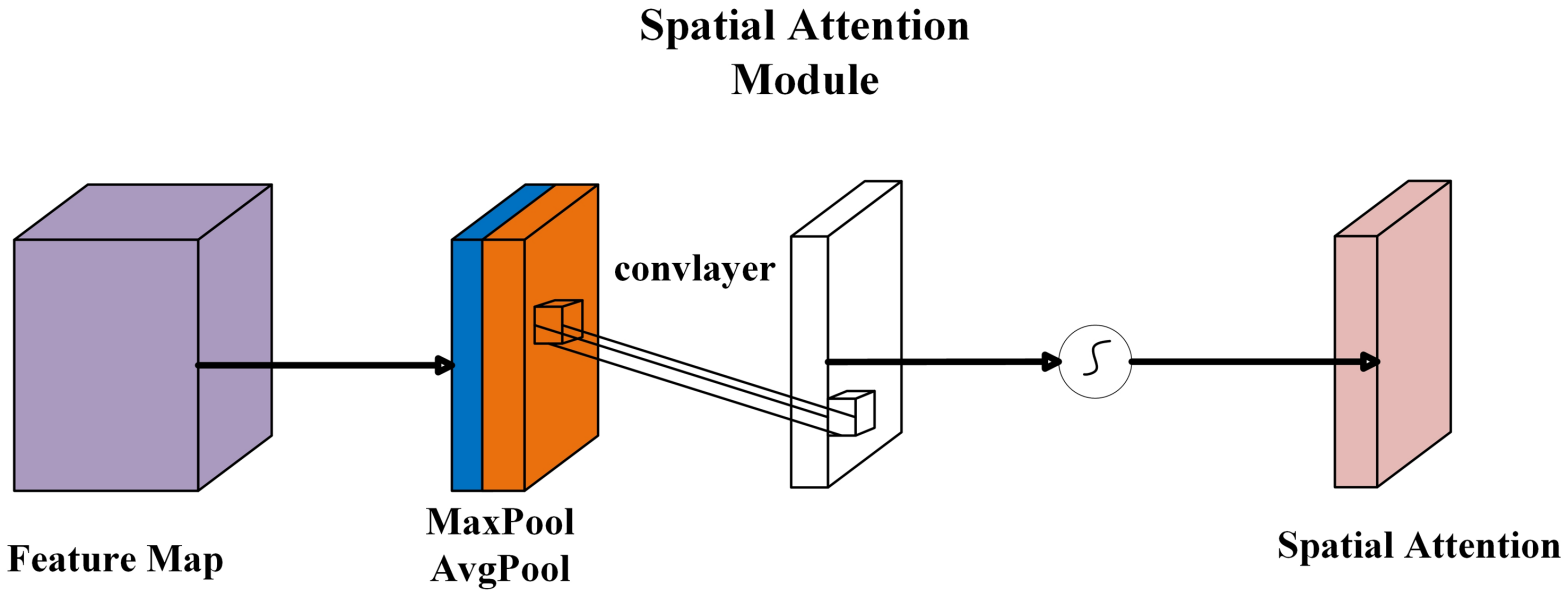
Spatial attention mechanism.

Spatial attention is a technique designed to improve the ability of convolutional neural networks (CNNs) to focus on key regions when processing images. Traditional CNNs extract features by applying the same convolutional kernel across the entire feature map; however, this approach fails to capture important local information effectively. Spatial attention, on the other hand, assigns different weights to each spatial location in the input feature map, enabling the network to focus on key information regions during image processing, thereby improving network performance. A schematic diagram of the spatial attention mechanism is shown in Figure 9.

#### 2.3.3 Hybrid attention mechanism

**Figure 10:**
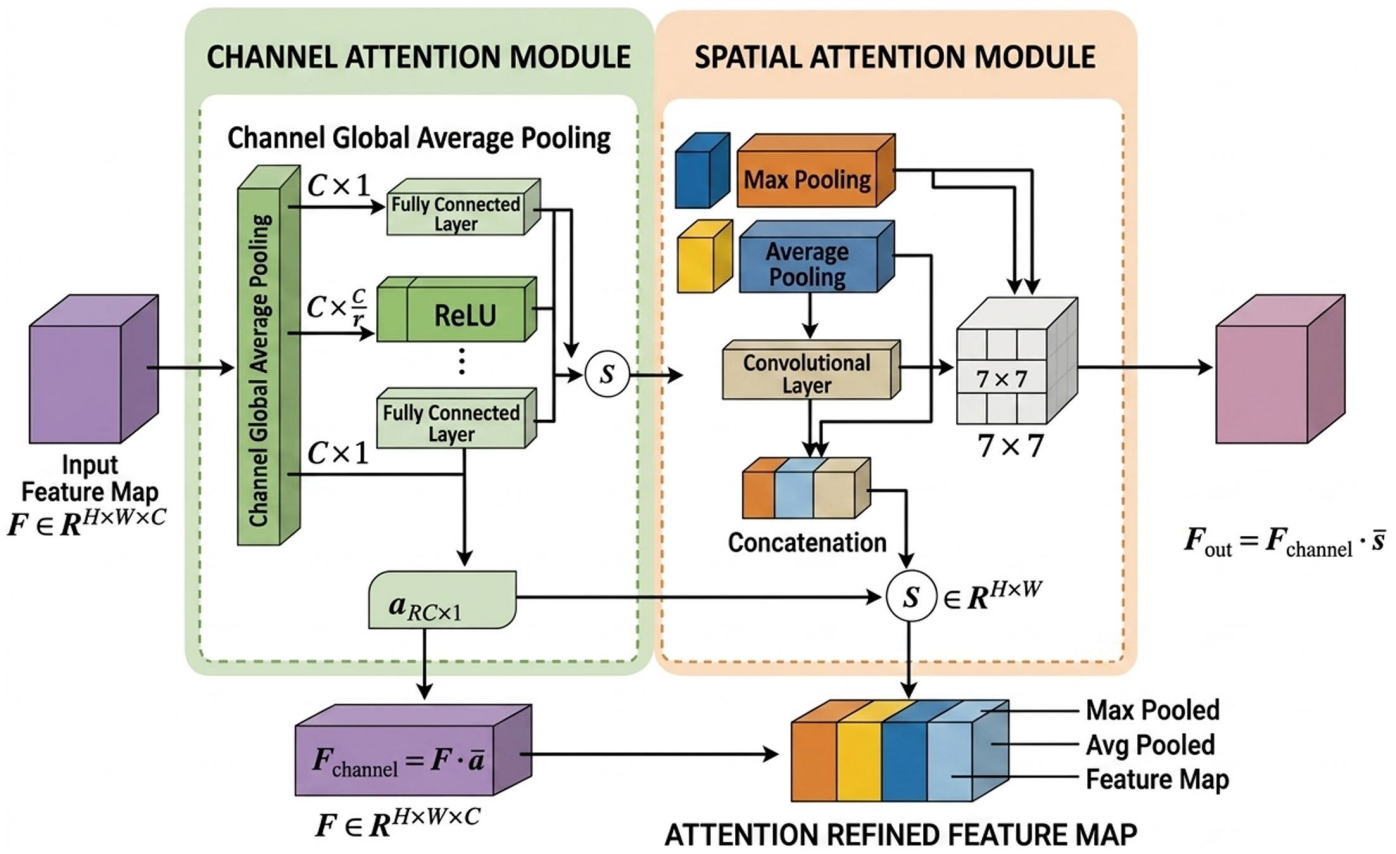
Hybrid attention mechanism.

The core idea of the hybrid attention mechanism is to simultaneously weight the feature map in both the channel and spatial dimensions. Specifically, it first uses a channel attention mechanism to select and weight important channels in the feature map, and then uses a spatial attention mechanism to weight each spatial location. This combination allows for finer control over the contribution of each element in the feature map, focusing on both channels containing key information and important regions in the image. The flowchart of the hybrid attention mechanism is shown in Figure 10.

The Integrated Attention Module improves a model’s performance by focusing on important features, using both channel attention and spatial attention mechanisms.

In the Channel Attention Module, the feature map $F\in {\mathbb{R}}^{H\times W\times C}$ (height H , width W , channels C ) is first processed with global average pooling. This reduces the spatial dimensions (height and width) to produce a vector $F_{\mathrm{avg}}$ , summarizing the information of each channel as shown in Equation (7):


$$F_{avg}=\frac{1}{H\times W}\overset{H}{\underset{i=1}{\sum }}\overset{W}{\underset{j=1}{\sum }}F\left(i\mathrm{,,}j\mathrm{,,}:\right)$$ (7)

This vector is then passed through a series of fully connected layers to generate channel-wise attention weights $\alpha_{\mathrm{channel}}$ as shown in Equation (8):


$$\alpha_{channel}=FC\left(ReLU\left(FC\left(FC\left(F_{avg}\right)\right)\right)\right)$$ (8)

These weights are used to scale the original feature map, emphasizing the important channels as shown in Equation (9):


$$F_{channel}=F\cdot \alpha_{channel}$$ (9)

In the Spatial Attention Module, the feature map F is processed through both MaxPooling and AvgPooling operations to capture the most important local features. This results in two maps, $F_{\max}$ and $F_{\mathrm{avg}}$ as shown in Equation (10):


$$F_{\max}=MaxPool\left(F\right),F_{avg}=AvgPool\left(F\right)$$ (10)

These maps are then concatenated and passed through a convolutional layer to generate a spatial attention map $S_{\mathrm{spatial}}$ as shown in Equation (11):


$$S_{spatial}=ConvLayer\left(concat\left(F_{\max},F_{avg}\right)\right)$$ (11)

This spatial attention map is used to scale the original feature map, emphasizing the important spatial regions as shown in Equation (12):


$$F_{spatial}=F\cdot S_{spatial}$$ (12)

Finally, the outputs from the channel and spatial attention modules are combined. The final attention-enhanced feature map $F_{\mathrm{out}}$ is obtained by element-wise multiplying the channel-attended and spatial-attended feature maps as shown in Equation (13):


$$F_{out}=F_{channel}\cdot F_{spatial}$$ (13)

### 2.4 Network architecture

**Figure 11:**
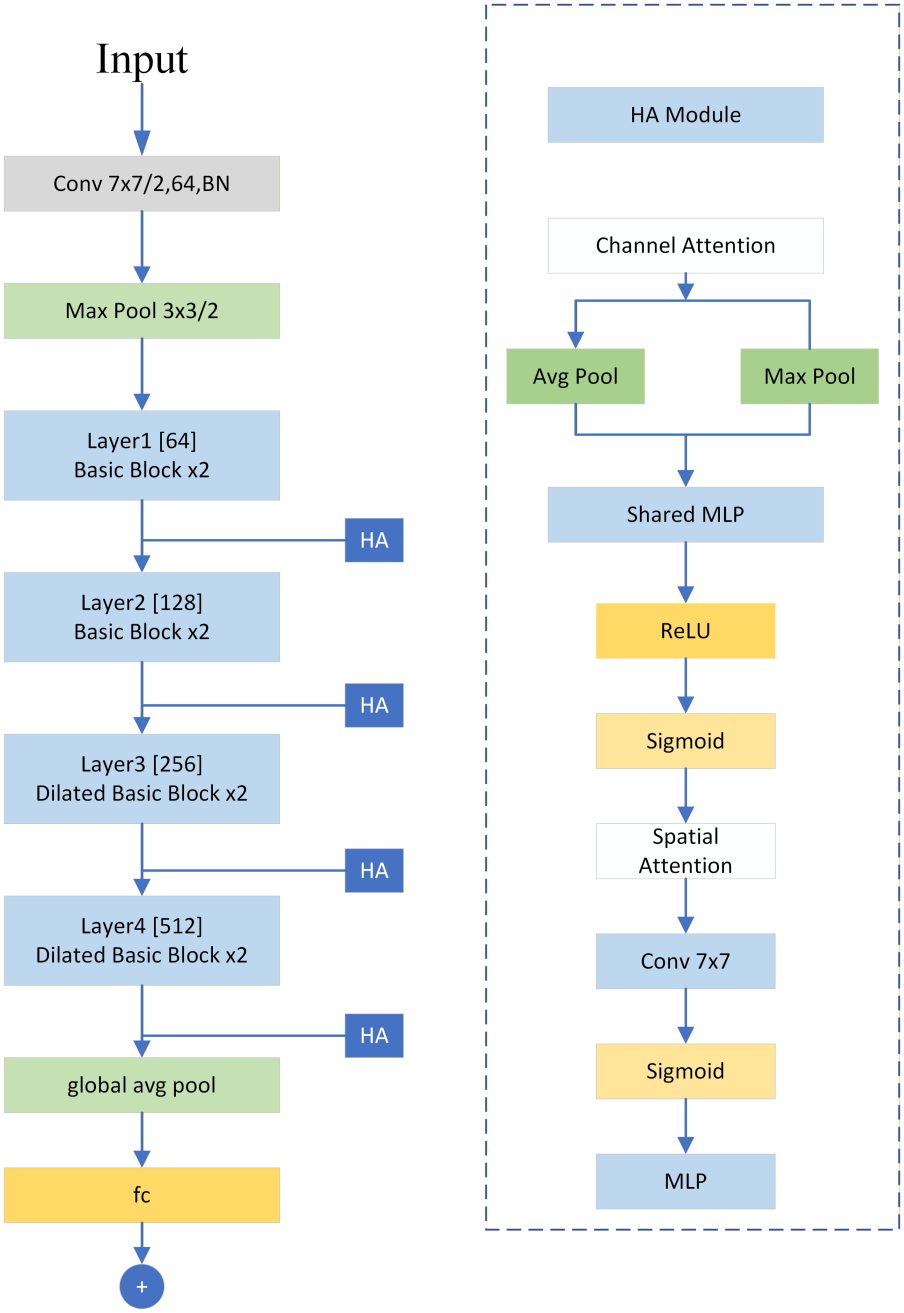
ResNet18-HA architecture.

In the field of abnormal fetal heart rate detection, the limited amount of data is a common problem. Using overly complex network model architectures can easily lead to overfitting and limit the model’s generalization ability. Therefore, this paper prioritizes the lightweight and high-performance ResNet-18 as the base framework, incorporating a hybrid attention mechanism into its original structure to further improve the targeting and effectiveness of feature extraction. The specific architecture of this model is shown in Figure 11.

## 3 Results

### 3.1 Experimental design

**Figure 12:**
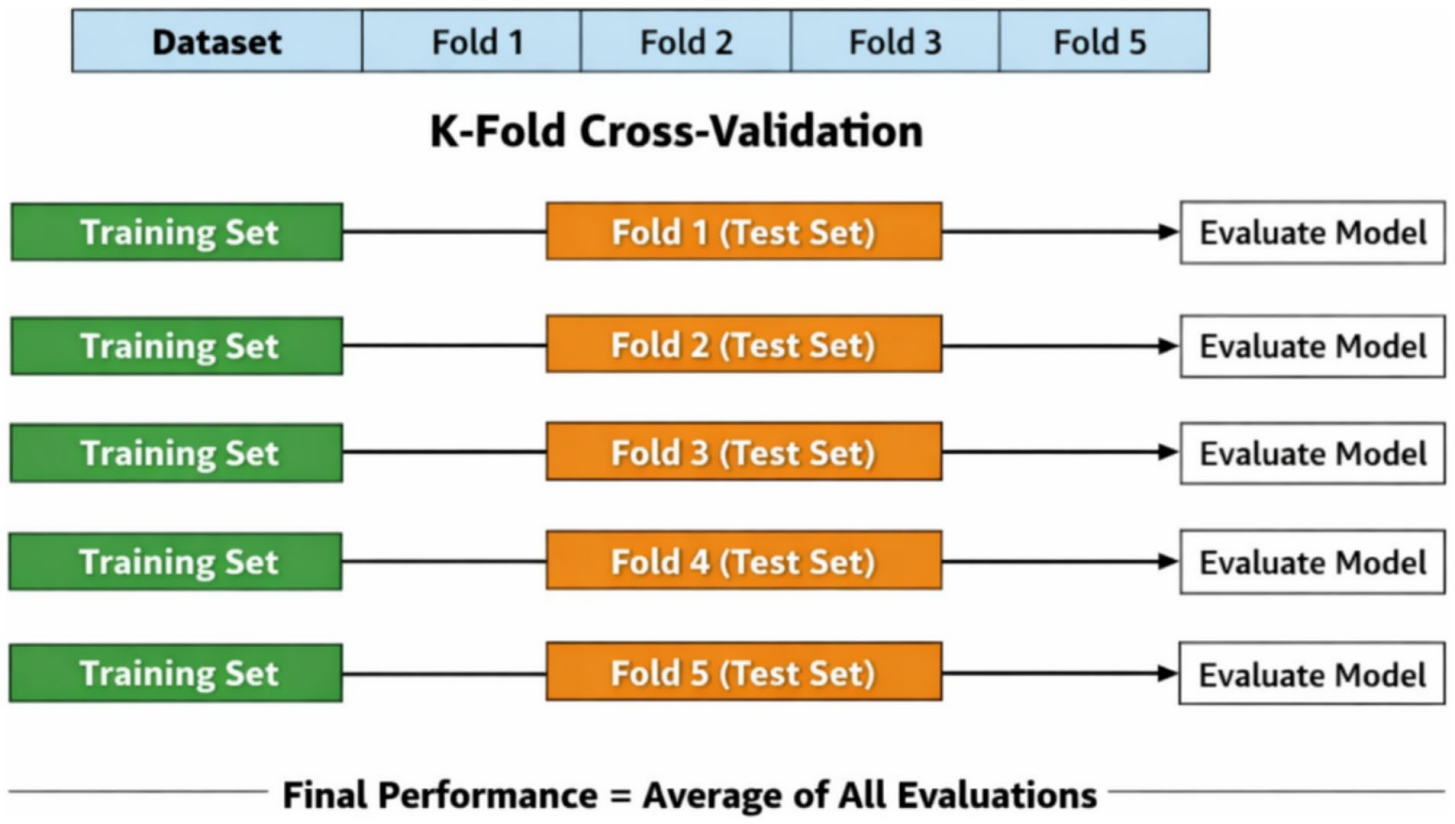
*K*-fold cross-validation.

Because the fetal heart rate monitoring data sample size is relatively limited, if only a single training set-test set partitioning is used, the model evaluation results are easily affected by the randomness of the data partitioning, thus reducing the reliability of the experimental conclusions. To make full use of the limited sample size and improve the stability of model performance evaluation, this paper uses the K-fold cross-validation method for model training and evaluation. A schematic diagram of K-fold cross-validation is shown in Figure 12.

This study is based on PyTorch 2.4.1, using the deep learning framework to build deep learning network models. The graphics card used is the NVIDIA RTX A6000 with 48 GB of memory. Due to the relatively small dataset, the model trained with a single iteration is likely to suffer from significant discrepancies in the division of training and testing sets. This article decides to use k-fold cross-validation to increase the reliability of the experimental results. The dataset D is divided into K subsets {D1, D2, …, DK}, from which K-1 subsets are used for training and the remaining one is used for testing, conducting the model training and testing. This process is repeated K times, ensuring that each subset is used for testing once. The final model is evaluated by averaging the K validation results. This article also applies transfer learning methods, adjusting the pre-trained base model weights to fine-tune the training, significantly reducing training time.

### 3.2 Evaluation indicators

We use a confusion matrix to evaluate the experimental results, as shown in Table 3. In this evaluation framework, a normal fetus is defined as a positive class, and an abnormal fetus is defined as a negative class. Based on this setting, the model prediction results are defined as follows: a correct prediction of a normal fetus is called a true positive (TP); a incorrect prediction of an abnormal fetus as a positive class is called a false positive (FP); a correct prediction of an abnormal fetus as a negative class is called a true negative (TN); and a incorrect prediction of a normal fetus as a negative class is called a false negative (FN).

**Table 3:** Confusion matrix.

Actual	Positive	Negative
Positive	TP	FN
Negative	FP	TN

Based on the confusion matrix, this paper uses Precision, Recall, F1-score, Specificity and Accuracy to evaluate the experimental results, as shown in the following formulas as shown in Equation (14–18):


$$Precision=\frac{TP}{TP+FP}$$ (14)

$$Recall=\frac{TP}{TP+FN}$$ (15)

$$F1-score=\frac{2\cdot Precision\cdot Recall}{Precision+Recall}$$ (16)

$$Acc=\frac{TP+TN}{TP+FN+FP+TN}$$ (17)

$$\mathrm{Specificity}=\frac{TN}{TN+FP}$$ (18)

The loss function adopts the cross entropy loss function, which is as shown in Equation (19):


$$L\left(y,\hat{y}\right)=-\left(y\log\left(\hat{y}\right)+\left(1-y\right)\log\left(1-\hat{y}\right)\right)$$ (19)

Because time-series graphs have a certain temporal sequence, data augmentation methods such as flipping and cropping used in other computer vision tasks cannot be employed. The hyperparameters of the model trained in this paper are shown in Table 4.

**Table 4:** Hyperparameter list.

Hyperparameter	Value
K value	5
Epochs	25
Learning rate	0.001
Momentum	0.9
Weight decay	0.0001

### 3.3 Results analysis

**Figure 13:**
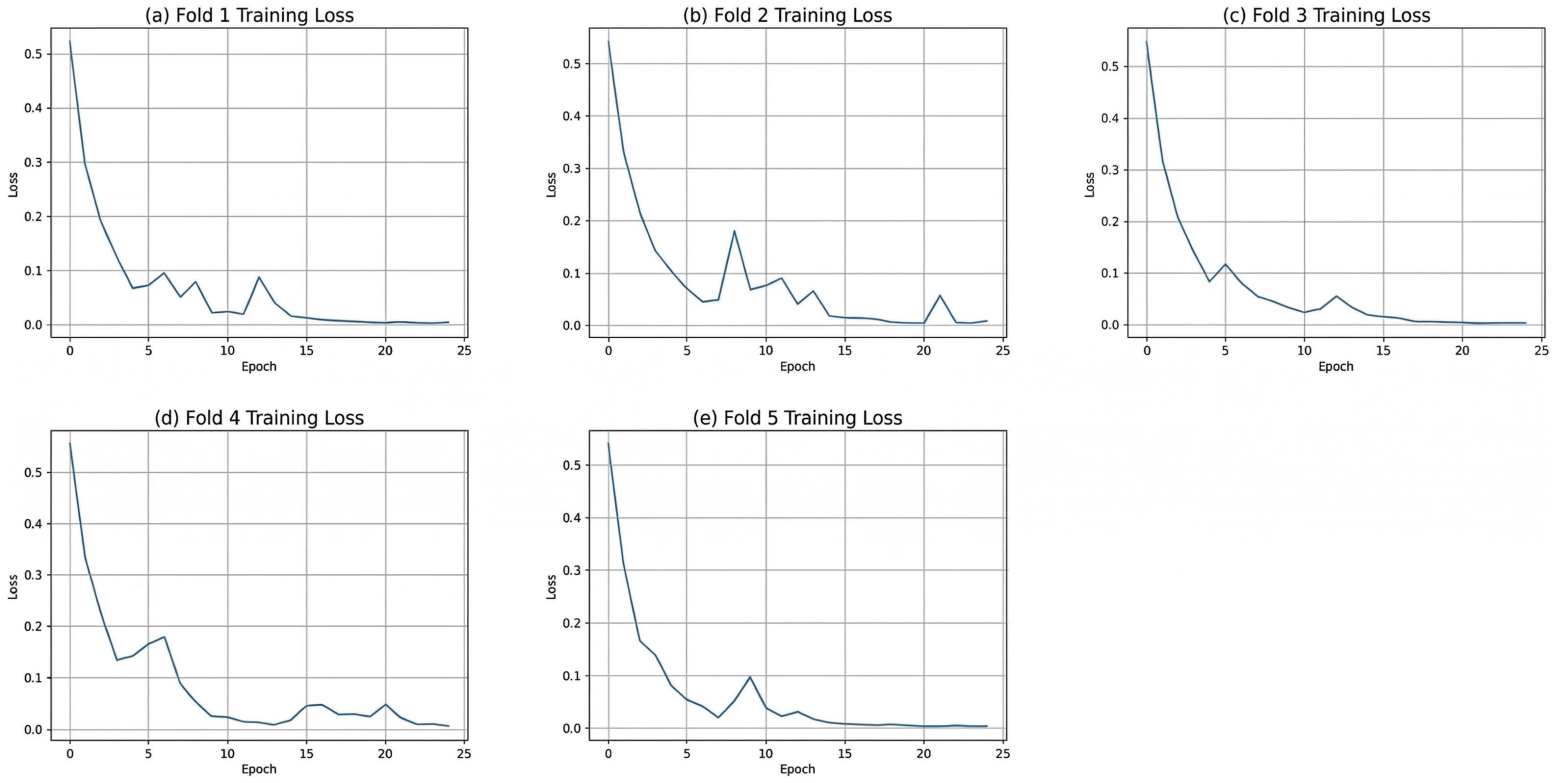
Loss value during training. **(a)** Fold 1 training loss, **(b)** Fold 2 training loss, **(c)** Fold 3 training loss, **(d)** Fold 4 training loss, **(e)** Fold 5 training loss.

Figure 13 shows the change in the loss function during training. These graphs illustrate the training process of the model in five-fold cross-validation, with each subplot representing the change in training loss for one fold over training epochs. As can be seen from the graphs, the training loss for all folds exhibits a rapid decline phase, especially in the first few epochs. The loss curve for each fold decreases rapidly within the first five epochs, typically indicating that the model effectively fits the data in the early stages of learning. However, after the loss decreases to a lower level, the loss curves for each fold tend to plateau, indicating that the model begins to converge in the later stages of training.

**Figure 14:**
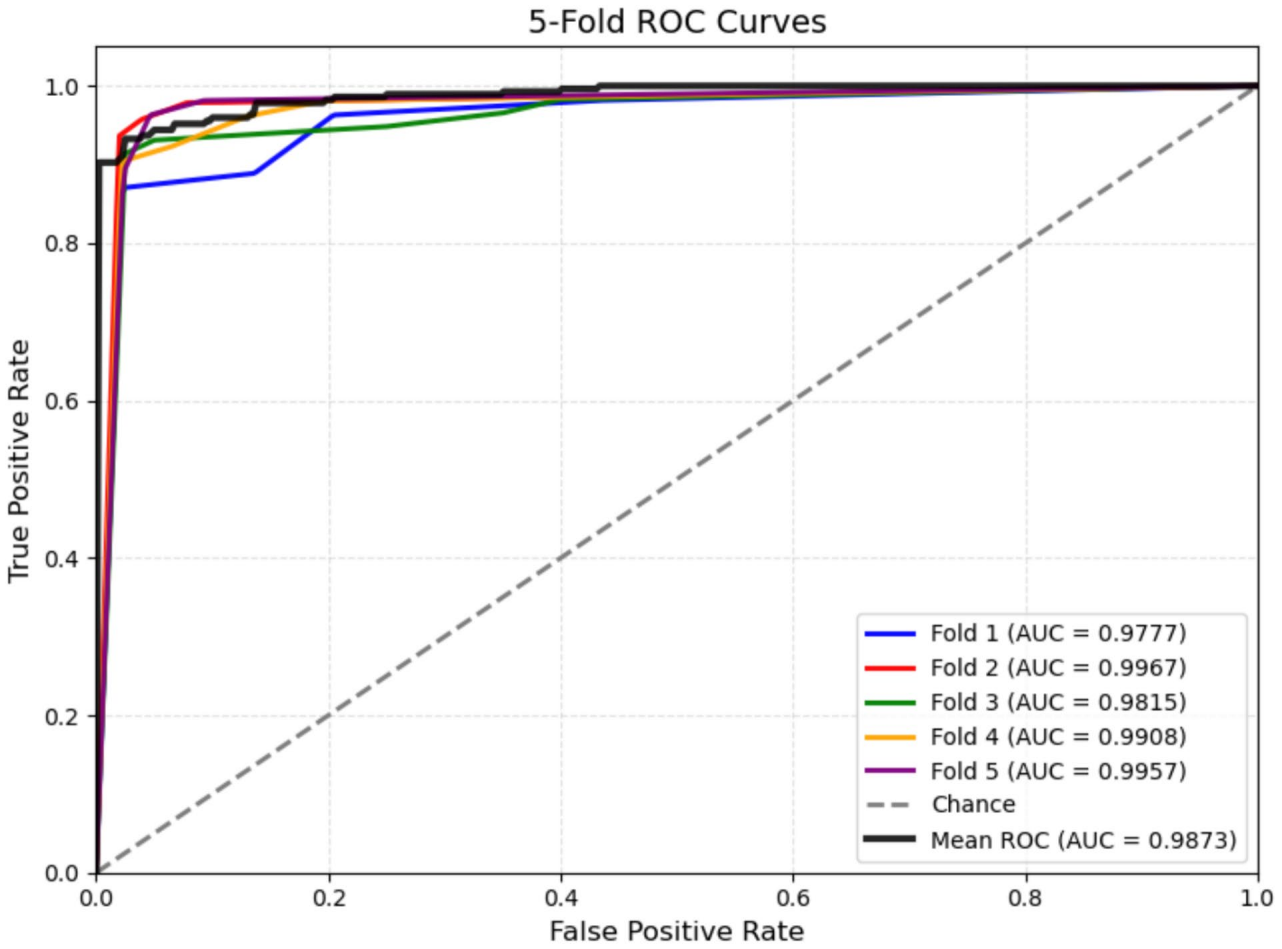
ROC curve.

Figure 14 presents the receiver operating characteristic (ROC) curves of the proposed model under five-fold cross-validation. The ROC curves of all five folds were consistently close to the upper-left corner, indicating that the model achieved excellent discrimination ability between normal and pathological fetal states across different data splits. The area under the ROC curve (AUC) values for Fold 1 to Fold 5 were 0.9777, 0.9967, 0.9815, 0.9908, and 0.9957, respectively, with a mean AUC of 0.9873. These results demonstrate that the proposed model maintained high classification performance and good robustness throughout the cross-validation process.

Among the five folds, Fold 2 and Fold 5 achieved the highest AUC values, both exceeding 0.99, while Fold 1 showed a slightly lower but still strong AUC of 0.9777. Although slight variations were observed among different folds, all AUC values remained above 0.97, suggesting that the model performance was stable and not highly sensitive to the specific partitioning of the dataset.

**Figure 15:**
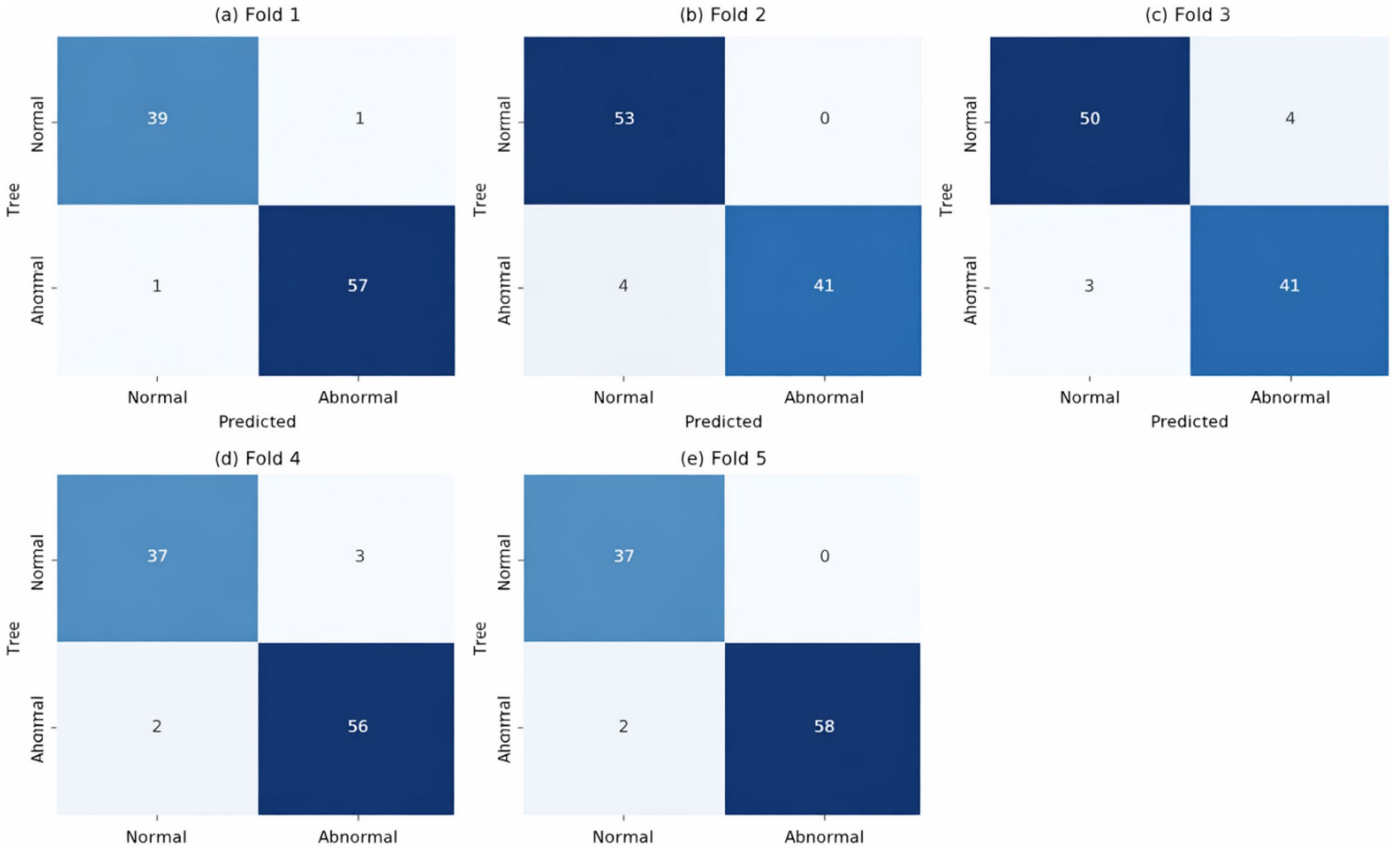
Confusion matrix. **(a)** Fold 1, **(b)** Fold 2, **(c)** Fold 3, **(d)** Fold 4, **(e)** Fold 5.

Figure 15 shows the confusion matrix of the model in five-fold cross-validation. Each subplot corresponds to a fold (Fold 1 to Fold 5) and is used to evaluate the model’s classification performance. The model generally performs well across all folds, accurately predicting most normal and abnormal samples. In fold 1, 39 normal samples were predicted as normal, and 57 abnormal samples were predicted as abnormal, but there were also a few false positives (1 normal sample predicted as abnormal) and false negatives (1 abnormal sample predicted as normal). Fold 2 showed high accuracy, with 53 normal samples correctly predicted as normal, and abnormal sample predictions were also relatively accurate, with 4 abnormal samples mispredicted as normal. Folds 3 and 4 showed a similar pattern, with relatively accurate predictions of both normal and abnormal samples, and few false positives and false negatives. In fold 5, the prediction of normal samples was almost perfect, with all normal samples predicted as normal, while the accuracy of predicting abnormal samples as abnormal was high.

To further analyze the mechanism of the hybrid attention mechanism at different feature levels, this paper employs ablation experiments, introducing the HA module into different layers of the network and systematically comparing the classification performance of the corresponding models. Considering the significant differences in features extracted by convolutional neural networks at different layers—shallow features focusing on local texture and edge information, while deeper features represent more global semantic information—comparing the effects of introducing HA at different layer positions helps reveal the differences in the role of the attention mechanism at different feature levels and its impact on the overall model performance, as shown in Table 5. Here, Model 1 to Model 15 denote the models with the HA mechanism introduced at different layers.

**Table 5:** Results of the ablation study.

Model	Layer 1	Layer 2	Layer 3	Layer 4	Acc (%)	Recall (%)	Precision (%)	F1-Score (%)	Specificity (%)	Parameters (M)
Baseline					93.88	93.83	93.98	93.87	93.93	11.17
Model 1				✓	97.94	98.33	97.44	97.84	97.55	11.21
Model 2			✓		94.90	94.79	95.45	94.87	95.01	11.18
Model 3			✓	✓	95.92	95.87	96.03	95.91	95.97	11.21
Model 4		✓			89.80	89.87	90.01	89.79	89.73	11.17
Model 5		✓		✓	93.88	93.92	93.92	93.88	93.84	11.21
Model 6		✓	✓		91.84	91.87	91.87	91.84	91.81	11.21
Model 7		✓	✓	✓	89.80	89.92	90.32	89.79	89.68	11.22
Model 8	✓				91.84	91.92	92.06	91.83	91.76	11.17
Model 9	✓			✓	93.88	93.87	93.87	93.87	93.89	11.21
Model 10	✓		✓		91.84	91.87	91.87	91.84	91.81	11.18
Model 11	✓		✓	✓	93.88	93.75	94.64	93.84	94.01	11.22
Model 12	✓	✓			93.88	93.83	93.98	93.87	93.93	11.18
Model 13	✓	✓		✓	89.80	89.92	90.32	89.78	89.68	11.21
Model 14	✓	✓	✓		95.92	95.92	95.92	95.92	95.92	11.19
Model 15	✓	✓	✓	✓	94.90	94.87	94.93	94.89	94.93	11.22

Table 5 summarizes the results of the ablation experiments after introducing the hybrid attention mechanism (HA) at different convolutional layer locations. The baseline model without HA achieved an accuracy of 93.88%, a recall of 93.83%, a precision of 93.98%, an F1-score of 93.87%, and a specificity of 93.93%. After incorporating HA, the performance of different model configurations varied markedly, indicating that the effectiveness of the attention mechanism depended strongly on the insertion location rather than being uniformly beneficial. Across all configurations, accuracy ranged from 89.80 to 97.94%, F1-score ranged from 89.78 to 97.84%, and specificity ranged from 89.68 to 97.55%, further demonstrating that the placement of HA had a substantial impact on both positive and negative sample discrimination.

Among the single-layer insertion strategies, introducing HA at the deep feature stage produced the most pronounced improvement. Specifically, Model 1, which introduced HA only in the deep layer, achieved the best overall performance, with an accuracy of 97.94%, a recall of 98.33%, a precision of 97.44%, an F1-score of 97.84%, and a specificity of 97.55%. Compared with the baseline model, Model 1 improved accuracy by 4.06%, F1-score by 3.97%, and specificity by 3.62%, confirming that introducing attention at the deep semantic feature stage can substantially enhance the model’s discriminative capability. In contrast, models that introduced HA only in shallow or intermediate layers, such as Model 4 and Model 8, showed limited or even negative effects. For example, Model 4 exhibited an accuracy of 89.80%, an F1-score of 89.79%, and a specificity of 89.73%, all of which were clearly lower than those of the baseline model. This finding suggests that directly introducing attention mechanisms at low-level feature stages may interfere with the extraction of basic texture and edge information, thereby impairing classification performance.

For the multi-layer HA combinations, the experimental results showed that the effects of attention insertion across different layers were not simply additive. Some mid-to-deep combination models, such as Model 3 and Model 14, achieved relatively stable performance. Model 14 obtained 95.92% for accuracy, recall, precision, F1-score, and specificity, outperforming the baseline model overall, but still remaining inferior to Model 1. By contrast, when HA was introduced into too many layers simultaneously, model performance tended to decline. For example, Models 7 and 13 both achieved an accuracy of 89.80%, while their F1-scores were 89.79 and 89.78%, respectively, with both models showing a specificity of 89.68%. These results suggest that excessive attention modules may introduce feature redundancy or suppress key discriminative information, ultimately reducing model effectiveness.

**Figure 16:**
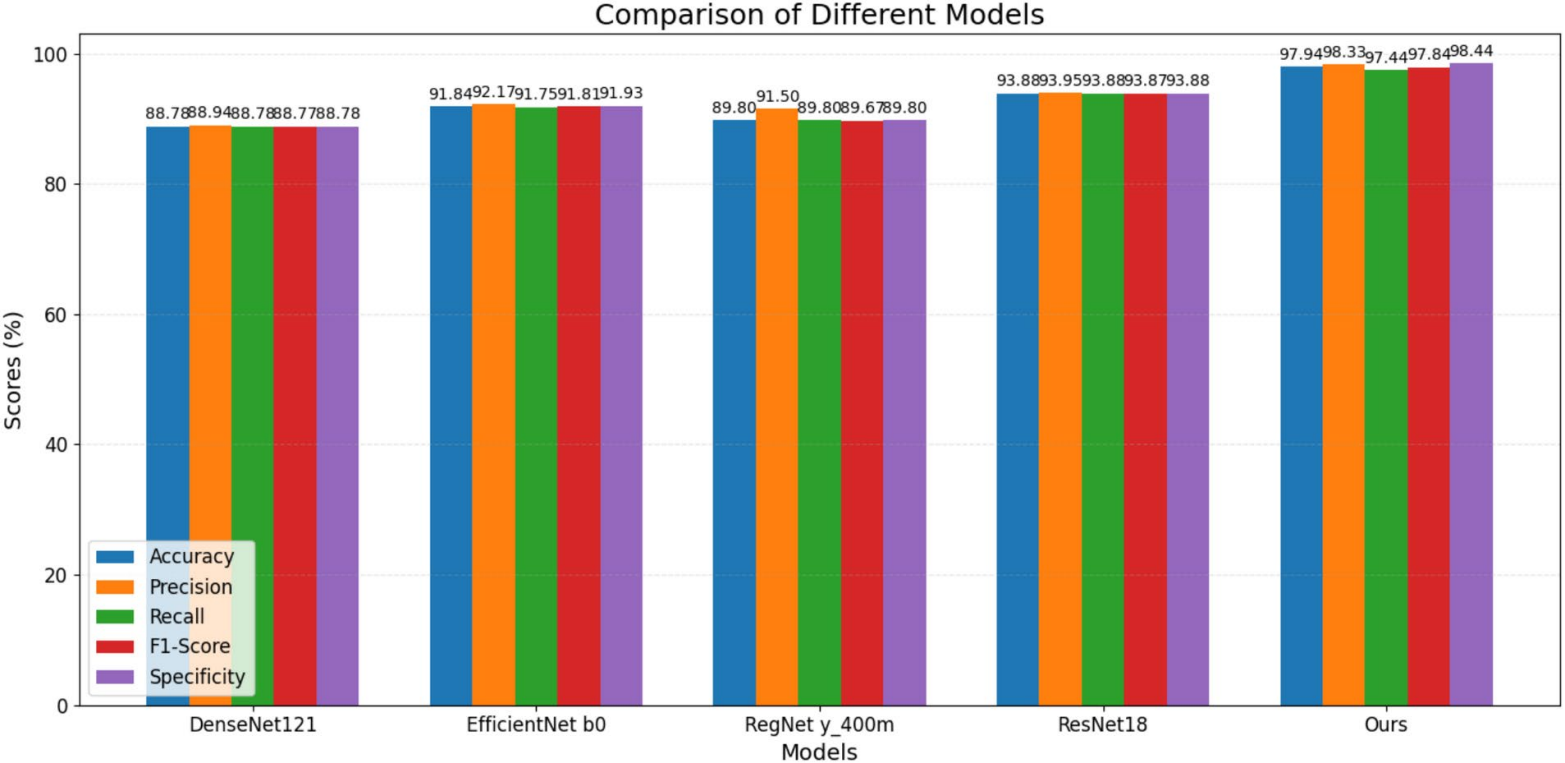
Comparison of different lightweight models.

Figure 16 shows a comparison between the model presented in this paper and other lightweight models.

Table 6 compares the results with previous work. Due to differences in data quality and the distribution of normal and anomalies, the evaluation indicators used are also different.

**Table 6:** Comparison with existing methods.

References	Dataset used	Sample size	Evaluation indicator			Method	Performance (%)
			AUC	ACC	F1		
Krupa, 2011 (22)	Private	90		✓		SVM	87
Spilka, 2014 (14)	Private	217			✓	NB, SVM, DT	71.5
Dash, 2014 (23)	Private	83			✓	GM, NB	69.9
Stylios, 2016 (24)	CTU-UHB	552	✓			LS-SVM	72.81
Li, 2018 (25)	Private	4,473		✓		CNN	93.24
O’sullivan, 2021 (26)	CTU-UHB	552	✓			ARMA-SVM	86
Ours	Private	489	✓	✓	✓	ResNet18 + HA	98.73/97.97/97.84

## 4 Discussion and prospect

In recent years, many scholars have explored machine learning and deep learning methods in fetal heart rate classification tasks. We propose a new research approach by introducing computer vision technology and utilizing a specific dataset from Chinese hospitals, combined with relevant computer vision models, to interpret electronic fetal heart rate monitoring signals from an image perspective. We achieved a high classification accuracy.

This study has the following limitations that need to be noted: First, the limitations of the dataset size and geographical origin (single center in Southwest China) may lead to insufficient generalization ability of the model to specific populations (such as obese pregnant women); second, although the HA attention module significantly improves model performance, the small amount of data may result in insufficient generalization ability; third, due to the lack of publicly available bimodal fetal heart rate monitoring benchmark datasets, existing comparative experiments can only be compared with single-modal methods and traditional machine learning baselines, and this benchmark difference may affect the objectivity of performance evaluation; finally, the current field of fetal heart rate monitoring lacks standardized signal preprocessing and annotation specifications, making it difficult to directly compare results from different studies. These limitations suggest that future research should focus on multi-center large-sample validation, computational efficiency optimization, and the establishment of standardized benchmarks. Although CTU-UHB and other public datasets were discussed as important benchmarks in the field, the present study did not perform direct external validation on these datasets. Therefore, the generalizability of the proposed model across different populations, acquisition settings, and annotation conventions has not yet been fully established. This should be considered when interpreting the performance advantage of our model over previously reported methods. In particular, because prior studies were conducted on different datasets with different class distributions, labeling criteria, and signal quality, cross-study comparisons should be interpreted cautiously rather than as definitive evidence of superior generalization. Future work will focus on multicenter validation and cross-dataset testing to further assess model robustness and external applicability.

To validate the model’s applicability in high-risk pregnancies, future plans include conducting specific validations for three high-risk subgroups: gestational diabetes, preeclampsia, and fetal growth restriction. A stratified sampling strategy will be used to ensure the representativeness of each subgroup, with a focus on monitoring the model’s ability to identify pathologically specific patterns (such as reduced accelerations in the gestational diabetes group and variable deceleration in the preeclampsia group).

## Data Availability

The data involves patient privacy, and a data confidentiality agreement has been signed with the hospital. Requests to access the datasets should be directed to XW, 1421603896@qq.com.
